# Hypothyroidism in Carcinoma of the Tongue with Adjuvant Treatment

**DOI:** 10.5041/RMMJ.10290

**Published:** 2017-07-31

**Authors:** Sabita Kumari, Jonathan Gondi, Hemantkumar Nemade, L. M. Chandra Sekhara Rao, Deleep Gudipudi, T. Subramanyeshwar Rao

**Affiliations:** 1 Department of Oral and Maxillofacial Surgery, Al Ameen Dental College, Bijapur, Karnataka, India; 2 Department of Surgical Oncology, Basavatarakam Indo-American Cancer Hospital and Research Institute, Hyderabad, India; 3 Department of Radiation Oncology, Basavatarakam Indo-American Cancer Hospital and Research Institute, Hyderabad, India

**Keywords:** Adjuvant treatment, chemo-radiotherapy, hypothyroidism, radiotherapy, tongue carcinoma

## Abstract

**Objective:**

The objective of this study was to evaluate the incidence of hypothyroidism with adjuvant treatment in oral tongue carcinoma patients treated primarily with surgery.

**Materials and methods:**

A retrospective review was carried out to analyze hypothyroidism incidence and its relation to adjuvant treatment (radiation/radio-chemotherapy) in oral tongue carcinoma after the primary surgical ablation and neck dissection. Hypothyroidism was analyzed in relation with dose of radiation, gender, and adjuvant treatment modality.

**Results:**

The study analyzed the patients who were treated between January 2012 and June 2015. Among 705 patients with carcinoma of the tongue treated primarily with wide local excision and neck dissection, 383 received adjuvant treatment. A total of 215 patients received radiation, and 168 received concurrent radio-chemotherapy. Of 378 patients, 78 developed hypothyroidism during follow-up: 27 patients received concurrent radio-chemotherapy, and the remaining 51 received only radiation. Lower neck received 40–48 Gy in 2 patients, 50 Gy in 74 patients, and 60–70 Gy and concurrent radio-chemotherapy in 27 patients. Median follow-up was 32 months. Hypothyroidism occurred in 21.5% of patients with squamous cell carcinoma of the oral tongue. The minimum period to develop hypothyroidism was 3 months in this study. Gender and adjuvant treatment were not found to be significant for the incidence of hypothyroidism.

**Conclusions:**

A significant number of patients with carcinoma of the tongue who receive adjuvant treatment will develop hypothyroidism, hence frequent monitoring of thyroid function is advised during follow-up.

## INTRODUCTION

Carcinoma of the oral cavity is among the top three types of cancers in India,^1^,^2^ and oral cancer accounts for about 30% of all types of cancer.^2^,^3^ Standard treatment of carcinoma of the tongue involves wide local excision with neck dissection, followed by adjuvant treatment depending upon stage and pathological risk factors. External beam radiotherapy (EBRT) is often associated with significant long-term morbidity, which includes chronic xerostomia and fibrosis of the soft tissues of the neck.^4^

The thyroid gland is the largest endocrine gland in human body. Thyroid hormones are crucial for metabolism, growth and development, energy expenditure, and the function of many organs.^4^ Radiation-induced thyroid complications, which include hypothyroidism, hyperthyroidism, Graves’ disease, benign adenoma, and even thyroid cancer, have been reported in the literature.^5^,^6^ Hypothyroidism is the most common complication of the radiation. The incidence of hypothyroidism varies with the risk factors such as surgery, radiation dose, volume of thyroid tissue irradiated, and duration of patient follow-up. Clinicians often overlook hypothyroidism as a complication of management of carcinoma in the oral cavity, and it has been pointed out that thyroid function is rarely evaluated at post treatment follow-up.^7^

The etiology of radiation-induced hypothyroidism remains unclear, but it is thought to be related to autoimmune reactions and parenchymal cell damage.^8^ The biological effects come from vascular damage and follicular cell function. Age and thyroid volume play an important role in the probability of occurrence of radiation-induced hypothyroidism. The minimal tolerance dose for the thyroid is considered to be 20 Gy when all or part of the gland is irradiated with conventional radiotherapy (RT).^5^ In a study by Mi Young Kim et al., the percentage of the thyroid volume absorbing >45 Gy was observed to be the best predictor of the development of hypothyroidism. The authors therefore suggest that 50% of the gland should receive at least 45Gy dosage for volumetric threshold of radiation-induced hypothyroidism.^9^

This study is a single-institution retrospective study to determine the incidence of hypothyroidism after RT and concurrent radio-chemotherapy (RT-CT) in oral tongue carcinoma after surgery.

## METHODS AND MATERIALS

### Patient Evaluation

The study is a single-institution retrospective analysis of 705 consecutive patients with a biopsy-proven squamous cell carcinoma of the oral tongue at the Basavatarakam Indo-American Cancer Hospital and Research Institute in Hyderabad, India between January 2012 and June 2015 who were treated with surgery, i.e. wide local excision with neck dissection (unilateral or bilateral as per primary tumor extent), followed by adjuvant RT and concurrent cisplatin-based chemotherapy according to the stage of the disease and histopathological risk factors discussed at a multidisciplinary tumor board. Out of a total of 705 patients operated for carcinoma of the tongue, 383 patients received adjuvant therapy. Five patients were excluded from the analysis as they had known thyroid disease before treatment. We had a total of 378 evaluable adult patients with carcinoma of the tongue for whom adjuvant RT or concurrent RT-CT was given, the thyroid gland was contoured, and the dose of radiation and hypothyroidism constraints were evaluated. Inclusion criteria included euthyroid patients, squamous histology, and oral tongue site. Patients were excluded if they had pre-existing thyroid disease or previous thyroid surgery, oral cavity sites other than the tongue, previous cancer treatment, and treatment with isotopes. Pretreatment assessment included history, general and physical examination, biopsy and endoscopic staging of tumor, viral screening, CT or MRI, chest X-ray, and dental evaluation. Positron emission tomography–computed tomography (PET CT) was carried out if required and CT of chest if at all indicated. Routine preoperative serum thyroid-stimulating hormone (TSH) analysis was not done for all patients, only in those cases where there was clinical suspicion for thyroid dysfunction.

### Endocrine Assessment

Thyroid function was determined by measuring the value of serum TSH. Patients were defined as euthyroid if they had a normal TSH measurement. The biochemistry and laboratory medicine department of our institute defined the normal range of TSH as 0.27–4.2 microIU/L by sandwich-type immunoassay. We defined hypothyroidism if the TSH value was greater than our institutional upper limit. The interval between the end of RT and first recorded abnormal TSH value was defined as the time onset for developing hypothyroidism. Patients with normal serum TSH levels were classified as normal; those with high serum TSH were classified as hypothyroid.

### Radiation Treatment

For the radiation dose to be delivered, thermoplastic shells were individually molded to cover the head and neck area for immobilization.^10^ Computed tomographic simulation was performed in all patients and treatment planned using a beam of 6-MV photons with 2 Gy per fraction, once daily at 5 days per week with linear accelerator with two opposite equally weighted lateral portals plus one anterior portal. An anterior portal was treated to a dose of 50 Gy. Lateral portals were cone downed after 40 Gy and continued to a dose of 60–66 Gy depending on final histopathology. External beam radiotherapy was stopped for grade 4 mucositis.

### Chemotherapy

Patients who had positive margins, extranodal spread, and N2 or N3 disease received weekly chemotherapy with cisplatin 40 mg/m^2^ concurrently along with EBRT. Chemotherapy was withheld for grade 4 mucositis.

### Follow-Up

Patients were kept under follow-up and examined every 6–8 weeks for the first year, every 2–3 months during the second year, every 3–6 months during the third year, and annually thereafter. Routine testing of TSH was monitored in all patients after the completion of EBRT at 3–6 months, 9 months, 1 year, and thereafter.

### Statistical Analysis

The relative significance of variables such as age, gender, and radiation dose treatment, including RT and concurrent RT-CT, was obtained, and correlations between these variables were obtained by using a statistical test, i.e. chi-square test. Correlations between the incidence of RT and concurrent RT-CT were obtained by chi-square test.

## RESULTS

We performed this study to evaluate the incidence of thyroid hypofunction after adjuvant conventional RT and RT-CT for oral tongue carcinoma. A total of 705 patients were treated with surgery for carcinoma of the tongue; 383 received RT, out of which 5 patients had pretreatment hypothyroidism hence were excluded from the analysis (Table 1). Altogether 215 patients received adjuvant radiation, while 168 patients received concurrent RT-CT; 78 patients developed hypothyroidism. A total of 51 patients with radiation and 27 patients with RT-CT developed hypothyroidism. In all these patients, serum TSH was obtained for follow-up. The study population shows that the patients with a longer follow-up had an increasing incidence of hypothyroidism after radiation, and they were put on levothyroxine. The earliest incidence of hypothyroidism occurred within 3 months in one patient. The incidence of radiation-induced hypothyroidism was 23.5% after RT and 16% after concurrent RT-CT, which was not statistically significant.

**Table 1:** Demographic, Clinical Characteristics of Study Subjects (*n*=378).

Variable		*n*	Hypothyroid Subjects	Percentage	*P* value
Gender	Male	274	58	21.16%	0.29
	Female	104	20	19.23%	

Age	25–40 years	124	28	22.58%	0.19
	41–65 years	222	48	21.16%	
	66–80 years	32	2	6.25%	

Treatment Modality	Radiotherapy	210	51	24.28%	0.60
	Concurrent radio-chemotherapy	168	27	16.07%	

## DISCUSSION

Before 1970, hypothyroidism was rarely reported following the treatment of head and neck cancer. Clinicians at that time were unaware of this association, and the thyroid gland was considered a radioresistant organ.^11^ It has been suggested that as there are few radiosensitive mitotic cells in the thyroid gland a prolonged interval is required for a sufficient number of cells to die following exposure to RT before there is any clinical manifestation.^8^ Various previous studies reveal that females have a higher incidence of hypothyroidism after surgery and RT. Posner et al. described this relationship in 1984,^12^ as did Vrabec and Heffron in 1981.^13^ Because the rate of spontaneous hypothyroidism is also much higher in females, this indicates a lower thyroid reserve in this population.^11^

Hypothyroidism represents the most frequent thyroid-related effect in patients treated with RT for head and neck cancers (20%–30%), but in clinical practice the dose to the thyroid gland and pre- and posttreatment levels of thyroid hormones have not yet been assessed.^5^ In our analysis, the incidence of hypothyroidism evaluated after adjuvant therapy (RT and concurrent RT-CT) was 21.5%, which is comparable with the results of historical series, i.e. in the range 20%–30%.^4^

The combination of surgery and radiation might facilitate the appearance of hypothyroidism following RT, and this was indicated by multivariate analysis by Tell et al. and by Grande et al.^14^,^15^ In their studies the dose of radiation to the lower neck had a significant effect on the incidence of hypothyroidism. Radiation-induced hypothyroidism usually manifests after a median interval of 15 months (range 7–32 months), which is important when defining a minimum follow-up period to assess for radiation-induced hypothyroidism. The etiology for the thyroid dysfunction is represented by vascular damage, parenchymal damage, and autoimmune reactions, and the effects of radiation on blood vessels are important as late effects of radiation to many different tissues and organs are mediated to some extent by effects of radiation on the vascularture.^7^

In a study by Weissler and Bery, neck dissection, gender, age, hyperfractionated RT, and the use of chemotherapy were not related to the development of an elevated TSH.^16^ Few studies have examined the late incidence of hypothyroidism in patients treated with RT, and fewer still have attempted to assess the impact of chemotherapy. The treatment group as well as gender, age, race, tumor status, lymph node status, primary site, and total radiation dose were examined for any association with the development of hypothyroidism, and each was found to be insignificant.^17^ In our study we found no significant difference in the incidence of the hypothyroidism with RT or RT-CT in adjuvant treatment. In our study gender was not found to be significantly associated with the incidence of hypothyroidism. Compared to RT alone, concurrent chemotherapy had better 5 year locoregional control, progression-free survival, and overall survival, but no difference in distant metastases.^18^ Concurrent chemotherapy increases the risk of greater than or equal to grade 3 acute mucositis but not late toxicity.^18^ Our study also shows that concurrent chemotherapy has no additive effect on the incidence of hypothyroidism, which manifests as late toxicity.

## CONCLUSIONS

Radiation-induced hypothyroidism is a common complication of treatment of carcinoma of the tongue. Addition of chemotherapy has no additive effect. Serial serum TSH assessment after adjuvant therapy is essential to tackle hypothyroidism and improve quality of life.

## Abbreviations

EBRT: external beam radiotherapy
PET CT: positron emission tomography–computed tomography
RT: radiotherapy
RT-CT: radio-chemotherapy
TSH: thyroid-stimulating hormone.
